# Use of Apolipoprotein B in the Era of Precision Medicine: Time for a Paradigm Change?

**DOI:** 10.3390/jcm12175737

**Published:** 2023-09-03

**Authors:** Justine Cole, Rafael Zubirán, Anna Wolska, Ishwarlal Jialal, Alan T. Remaley

**Affiliations:** 1 Lipoprotein Metabolism Laboratory, Translational Vascular Medicine Branch, National Heart, Lung and Blood Institute, National Institutes of Health, Bethesda, MD 20814, USA; rafael.zubiran@nih.gov (R.Z.); anna.wolska@nih.gov (A.W.); alan.remaley@nih.gov (A.T.R.); 2 Department of Pathology and Internal Medicine, University of California-Davis, Sacramento, CA 95817, USA; kjialal@gmail.com

**Keywords:** cardiovascular disease, ASCVD, atherosclerosis, dyslipidemia, apolipoprotein B, LDL-C, non-HDL-C, residual risk, statin, PCSK9 inhibitor

## Abstract

Atherosclerotic cardiovascular disease (ASCVD) remains the leading cause of death worldwide and the risk of a major cardiovascular event is highest among those with established disease. Ongoing management of these patients relies on the accurate assessment of their response to any prescribed therapy, and their residual risk, in order to optimize treatment. Recent international guidelines and position statements concur that the plasma concentration of apolipoprotein B (apoB) is the most accurate measure of lipoprotein associated ASCVD risk. This is especially true for the growing number of individuals with diabetes, obesity, or the metabolic syndrome, and those on statin therapy. Most guidelines, however, continue to promote LDL-C as the primary risk marker due to uncertainty as to whether the greater accuracy of apoB is sufficient to warrant a paradigm shift. Recommendations regarding apoB measurement vary, and the information provided on how to interpret apoB results is sometimes insufficient, particularly for non-lipid specialists. Misinformation regarding the reliability of the assays is also frequently repeated despite its equivalent or better standardization than many other diagnostic assays. Thus, demand for apoB testing is relatively low, which means there is little incentive to increase its availability or reduce its cost. In this review, we examine the results of recent clinical outcomes studies and meta-analyses on the relative values of apoB, LDL-C, and non-HDL-C as markers of ASCVD risk. Although there is seemingly minimal difference among these markers when only population-based metrics are considered, it is evident from our analysis that, from a personalized or precision medicine standpoint, many individuals would benefit, at a negligible total cost, if apoB measurement were better integrated into the diagnosis and treatment of ASCVD.

## 1. Introduction

Despite recent treatment advances, atherosclerotic cardiovascular disease (ASCVD) remains the leading cause of death worldwide [1]. Secondary prevention is aimed at reducing the risk of a major adverse cardiovascular event (MACE) in patients with established ASCVD. It typically involves the use of high-intensity statins, often in conjunction with relatively expensive add-on therapies, such as proprotein convertase subtilisin/kexin type 9 inhibitors (PCSK9i) and bempedoic acid. Given the high stakes of over- and under-treatment, it is of great importance that the correct therapeutic decisions are made, which relies on using the most accurate markers of ASCVD risk.

It is well established that the trapping of apolipoprotein (apo) B-containing lipoproteins and retention of their cholesterol in the arterial wall is an early step in atherosclerotic plaque formation. Hence, the measurement of cholesterol in the blood emerged early on as a key ASCVD risk marker. Initially, total plasma cholesterol (TC) concentration was used, but, in the 1950s, investigators, like Gofman et al. [2] and Olson [3], found that only the apoB-containing lipoproteins are positively associated with coronary artery disease. The seminal discovery of the role of the low-density lipoprotein (LDL) receptor (LDLR) in lipoprotein metabolism and atherosclerosis by Brown and Goldstein in 1974 [4] further solidified the importance of LDL in the pathogenesis of ASCVD. Consequently, in 1988, the National Cholesterol Education Program (NCEP) promoted LDL cholesterol (LDL-C) as the primary target for therapy [5], continuing the tradition of emphasizing the lipoprotein cholesterol content in risk assessment. This coincided with the development of the first statin, lovastatin, which was the first effective and well-tolerated therapy for achieving substantial LDL-C reductions.

The attributes of apoB-containing lipoproteins that are frequently considered as being potentially relevant for understanding their atherogenicity are the type of lipoprotein, their cholesterol and triglyceride content, the particle size, and particle number. Numerous studies have recently established that the particle number of atherogenic lipoproteins (apoB-containing lipoproteins), and not their cholesterol content nor their type, is the most important attribute for determining ASCVD risk [6,7,8,9,10]. Given that all apoB-containing lipoproteins are atherogenic to varying degrees, and that apoB exists as a single copy on all of these lipoproteins, using apoB is a convenient way to measure the atherogenic particle number. 

In 2009, the AACC Lipoprotein and Vascular Diseases Division Working Group on Best Practices published a position statement describing why apoB is the best risk marker for clinical practice [6]. In 2013, the same group supported the adoption of apoB measurement in ASCVD risk assessment and favored treatment guidelines that utilized apoB [11]. Recently, an even stronger rationale exists for leveraging the benefits of apoB for ASCVD risk assessment, given the growing number of patients with obesity, type II diabetes mellitus, or the metabolic syndrome. These patients are known to have abnormal lipoprotein profiles, with high triglycerides (TGs), low high-density lipoprotein cholesterol (HDL-C), and elevated small, dense LDL (sdLDL) particle number, but normal or only slightly elevated LDL-C. This profile often leads to discordance between the LDL particle number (LDL-P), for which apoB is a close proxy, and LDL-C, and may lead to erroneous LDL-C-based therapeutic decisions [12,13]. The discordance between apoB and LDL-C is also of particular relevance in statin-treated patients, whose LDL-C and non-HDL-C are reduced to a greater extent than their LDL-P (apoB) [14,15,16]. 

The Canadian Cardiovascular Society has provided percentile equivalent apoB cut-points and treatment targets alongside those for LDL-C and non-HDL-C in their guidelines since 2003, and apoB is an insured test in all but one province in Canada [17]. The most recent European Society of Cardiology and European Atherosclerosis Society (ESC/EAS) guidelines also provide both secondary non-HDL-C and apoB targets, and state that apoB may be the preferred test in patients with hypertriglyceridemia [18]. The 2018 US Multisociety guideline for lipid management, however, recommends apoB only as a risk enhancer test in patients with an intermediate 10-year risk score, or as an optional secondary target for high-risk patients. They state that a TG of ≥2.3 mmol/L may be a relative indication to measure apoB [19]. 

In this review, we examine the evidence from the past 15 years on the relative value of apoB versus LDL-C and non-HDL-C as ASCVD risk markers. We focus on the more recent clinical outcomes trials that revealed how treating to apoB targets would improve clinical outcomes for a substantial number of individuals compared to LDL-C or non-HDL-C targets.

## 2. ApoB Biochemistry and Lipoprotein Metabolism

ApoB is a large hydrophobic protein that is present as a single copy on LDL [20] and triglyceride-rich lipoproteins (TRLs), including chylomicrons (CMs) [21], very low-density lipoproteins (VLDLs), and intermediate-density lipoproteins (IDLs) (remnant lipoproteins) [22]. There are two isoforms of apoB in circulation, apoB100 and apoB48, both of which are detected in apoB assays [20]. ApoB100 comprises 4563 amino acids and is synthesized in the liver [23]. After lipidation via the microsomal triglyceride transfer protein (MTP), apoB100 is secreted as the main structural protein on VLDL [20,24]. It also contains a positively charged ligand-binding domain for the uptake of LDL through the LDLR [23]. ApoB48 is synthesized in the small intestine and is secreted as the main structural protein on CMs, which carry dietary lipids to the lymph [21]. It’s length is about 48% that of apoB100, owing to a stop codon introduced during mRNA editing [25]. ApoB48 lacks the C-terminal, LDLR-binding domain of apoB100, and CM remnants are instead cleared via the binding of apoE. 

**Figure 1:**
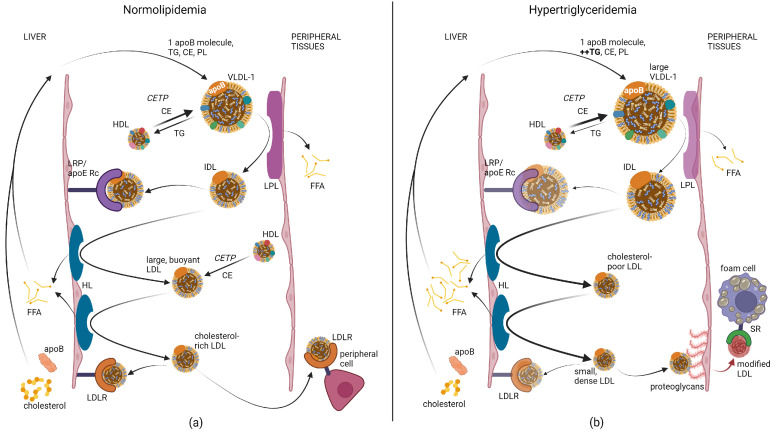
Triglyceride-rich lipoprotein metabolism. It was hypothesized that there may be 4 separate pathways for TRL metabolism [26], which depends on their size and apolipoprotein cargo. Here, two pathways are depicted. Arrow weight indicates flux through each pathway relative to “normal”. Translucence in the color of enzymes or receptors indicates decreased activity or affinity. (**a**) The hypothesized pathway for average sized VLDL and LDL. The weights of the arrows are almost all the same, to indicate “average” flux, but CETP transfers cholesteryl esters more readily than TGs. Intermediate-sized LDL is the preferred ligand for the LDLR. LDL also delivers cholesterol to steroid-producing tissues, which endocytose the particle via the LDLR. (**b**) The hypothesized pathway that predominates in hypertriglyceridemia. In hypertriglyceridemic patients, it seems that larger VLDL-1 is produced, that CETP may be rate-limiting for the transfer of TGs from VLDL-1 to HDL, and that LPL is less active. This results in larger, more TG-rich IDL species, which are not bound as readily by their hepatic receptors. Instead, they are processed by HL, which may have increased activity, resulting in small, cholesterol-poor LDL. This means that more FAs are delivered to the liver and less to the peripheral tissues. The LDLR also binds sdLDL less readily, whereas a change in apoB conformation and possibly a loss of sialic acid means that sdLDL binds readily to endothelial cell-surface proteoglycans. They are also more readily modified through oxidative processes. These damaged particles are removed by macrophages via scavenger receptors. Created with BioRender.com.

The metabolism of TRLs (Figure 1a) results in compositional changes and remodeling to other types of apoB-containing lipoproteins [26]. Both CMs and VLDLs are large lipoprotein particles (CM > 670 nm; VLDL 27–70 nm) that contain mostly TGs in their cores. Through lipolysis to fatty acids, these TGs are delivered to the peripheral tissues for either energy production or storage [20]. Alternatively, they may be transferred to other lipoproteins via the cholesteryl ester transfer protein (CETP) [20]. As TGs are depleted from their cores, phospholipids are removed from their shells, and the TRLs shrink to form remnant particles. As they shrink, the increasing surface tension [27] causes some of the exchangeable apolipoproteins, such as apoC-II and apoE, that modulate lipoprotein metabolism and cellular uptake, to dissociate from the TRL remnants [28]. CM remnants are removed rapidly by the liver and have a half-life of approximately 10 min in circulation [29,30].

During VLDL metabolism, remnant particles become enriched in cholesteryl esters, which are transferred to them from HDL particles via CETP [20,24,31]. VLDL is converted to IDL and much of this is subsequently converted to LDL, which contains approximately six to seven times more cholesterol than TG [20,26]. LDL is removed by the liver, via the LDLR, and has a half-life in plasma of approximately 3 days [20,31]. Thus, there is approximately 10 times more apoB100 than apoB48 in a fasting plasma sample, and about 90% of the apoB in circulation is on LDL.

Owing to the small diameter of LDL particles, averaging about 20 nm, LDL can readily enter the vessel wall and become trapped by the extracellular matrix in the intima. Furthermore, sdLDL may have a higher affinity for clearance through this route than average-sized or large LDL [32]. Within the arterial intima, LDL may undergo modification through a number of mechanisms, including oxidation, glycation, carbamylation and nitration, some of which occur specifically on the apoB molecule [33,34]. Oxidized apoB increases the uptake of LDL by macrophages and other cells to induce foam cell formation [35], and some of the lipid modification products promote inflammation [33,34]. Together, these processes eventually lead to atherosclerosis [20,24,31]. Given that it roughly estimates the concentration of LDL particles in circulation, LDL-C is associated with ASCVD events [36]. Unlike cholesterol, however, apoB is not exchanged between lipoproteins, and there is thus a fixed amount of apoB per LDL particle [37]. Therefore, the concentration of apoB is a more accurate measure of atherogenic lipoprotein particle number, especially when the proportion of sdLDL is elevated. ApoB also provides more information regarding the risk of LDL entering the subendothelium and causing atherogenesis and is thus more strongly associated with ASCVD [38,39,40]. The other apoB-containing lipoprotein particles also become atherogenic once they undergo sufficient lipolysis and become small enough to enter the vessel wall.

The size of a lipoprotein particle is also a determinant of its cholesterol-carrying capacity [20]. For example, larger LDL particles (20–22 nm) carry more cholesterol than small LDL particles (19–20 nm) [41], and typically account for about 60–70% of total LDL-C. As depicted in Figure 1b, the transfer of TGs from large VLDL particles to HDL by CETP may be reduced during hypertriglyceridemia, resulting in TG-enriched IDL and LDL particles [26]. The combined action of lipoprotein lipase and hepatic lipase on these particles results in the generation of small, cholesterol-poor LDL particles [26]. This explains the classic type B phenotype commonly seen in hypertriglyceridemia, in which LDL-C is normal or only slightly elevated, whereas apoB is almost always elevated [13,39]. As will be discussed in more detail, many studies have shown that when apoB and LDL-C are discordant, apoB is the better ASCVD marker [9,42]. Non-HDL-C, which is a measure of the cholesterol on all apoB-containing lipoproteins, is less affected by this issue but, in most studies, it was also found inferior to apoB as an ASCVD biomarker [43].

## 3. Clinical Utility of ApoB in Primary Prevention

Statins, the main lipid-lowering therapy, inhibit hepatic cholesterol synthesis, reducing VLDL secretion and upregulating LDLR expression, leading to lower LDL-C levels [44]. Clear evidence supporting the currently recommended LDL-C treatment targets and percentage reduction strategies is somewhat lacking [45], however, resulting in discrepant recommendations by the various guidelines [17,18,19]. In addition, despite reaching low LDL-C treatment goals, a large proportion of patients still experience atherosclerosis progression or ASCVD events [46]. This phenomenon of residual risk suggests that a singular focus on LDL-C measurement with fixed population-based treatment goals is not optimal for many patients [47].

Several guidelines propose non-HDL-C or apoB as secondary treatment targets, as intensifying lipid-lowering therapy to achieve these secondary targets mitigates the residual risk [48]. Treatment goals for non-HDL-C and apoB were originally derived from the LDL-C targets (Table 1) [17,18]. The American College of Cardiology and American Heart Association (ACC/AHA) Multisociety guideline does not include these parameters in its primary ASCVD prevention strategy but incorporates non-HDL-C in its secondary prevention algorithm for managing high-risk patients [19]. The non-HDL-C cut-offs are arbitrarily set to 0.8 mmol/L above the LDL-C cut-offs. It is important to emphasize that this value is based on the Friedewald equation, which incorrectly assumes that the ratio of TG to VLDL-C is fixed at 2.2 [49]. In contrast, the ESC/EAS recommend either apoB or non-HDL-C for risk assessment and provide targets for both parameters for primary prevention. They further suggest that apoB may be the preferred marker in patients with hypertriglyceridemia, obesity, or type II diabetes mellitus, and promote it as an alternative to LDL-C for assessing ASCVD risk [18]. The Canadian Cardiovascular Society strongly recommends using non-HDL-C or apoB instead of LDL-C as the primary risk marker for ASCVD. They provide targets for both in their graphical algorithm, increasing the likelihood that clinicians will understand and use these measures [17]. Both the ESC/EAS and Canadian guidelines acknowledge the superiority of apoB over non-HDL-C, but neither unequivocally recommends that apoB be prioritized as the therapeutic target. 

From a pathophysiological perspective, apoB is likely to be superior to both LDL-C and non-HDL-C as a biomarker as it represents the total atherogenic particle concentration rather than simply the cholesterol content of these particles. This is important, as the cholesterol content varies greatly within and between particle types. For example, in 50% of individuals, either smaller, cholesterol-depleted LDL particles or larger, cholesterol-enriched particles predominate [50]. In these individuals, LDL-C would either underestimate or overestimate the LDL-P, respectively. The importance of this issue was validated clinically in numerous discordance analyses, including the Coronary Artery Risk Development in Young Adults study [51], the Women’s Health Study [52], the INTERHEART study [53], the Framingham Heart Study [54], and the Copenhagen general population study [55], all of which support the concept that, when discordant, the risk of ASCVD is more closely related to the concentration of atherogenic lipoprotein particles than to the amount of cholesterol they carry. Such discordance is common in hypertriglyceridemia [56], obesity [57], the metabolic syndrome and type II diabetes mellitus [58], all of which are becoming more common throughout the world. These high-risk individuals have predominantly small, dense cholesterol-poor LDL particles, which explains why they have relatively normal LDL-C that underestimates the ASCVD risk that is evidenced with their higher LDL-P or apoB.

Another important issue is that statins increase the proportion of sdLDL particles compared to large buoyant LDL (lbLDL) particles [14]. This is because the LDLR has greater affinity for cholesterol-rich lbLDL [15]. Thus, although statins reduce the concentration of all LDL particles, they have a disproportionately larger effect in reducing LDL-C, leading to an underestimation of risk, particularly in patients with high levels of sdLDL with a higher LDL-P [8,10,11]. This phenomenon is evident in the discordant responses of LDL-C and apoB to statin treatment, where statins induced an LDL-C decrease of around 34% with a concomitant decrease of only 24% in apoB [59]. 

To the best of our knowledge, there are no trials that specifically examined apoB as a therapeutic target. Instead, it was studied in meta-analyses comparing non-HDL-C and apoB, with some contradictory findings. The 2007 emerging risk factors collaboration (ERFC) meta-analysis showed that the hazard ratios (HRs) for ASCVD of non-HDL-C and apoB were similar through the quintiles [60]. There were, however, several limitations in this meta-analysis, such as inclusion of studies that used non-standardized methods to measure apoB. In addition, it included several studies that were not published and therefore could not be fully evaluated. In 2012, Boekholdt et al. performed a meta-analysis comprising eight randomized studies evaluating the evidence for LDL-C, non-HDL-C, and apoB in patients treated with statins. The HRs for cardiovascular events were virtually identical and clinically indistinguishable for residual risk (HRs of 1.13, 1.16, and 1.14 were calculated for LDL-C, non-HDL-C, and apoB, respectively) [61]. The power of this study to determine superior precision amongst these markers was called into question by Thanassoulis et al., however, who performed a meta-analysis in 2014 of the seven largest and most important placebo-controlled statin trials. They concluded that apoB reduction confers better ASCVD risk reduction per one standard deviation (SD) of apoB than per one SD reduction of LDL-C or per one SD reduction of non-HDL (39% vs. 30% vs. 32%, respectively) [59]. Furthermore, a 2011 meta-analysis, performed by the same group, of 12 independent reports, including 233,455 patients and 22,950 events, reported that the relative risk reduction (RRR) associated with a one SD decrease in apoB was 5.7% greater than that of non-HDL-C and 12% greater than that of LDL-C (RRRs of 1.43, 1.34, and 1.25 were calculated for apoB, non-HDL-C, and LDL-C, respectively). They also calculated the number of cardiovascular events that would be prevented among adult US residents using apoB, non-HDL-C, and LDL-C as risk markers in a NCEP Adult Treatment Plan-III-based prevention strategy. They found that using apoB as the primary ASCVD risk marker would prevent 800,000 more events over 10 years than using LDL-C [62]. 

More recently, two other studies have further supported the superiority of apoB over other ASCVD risk biomarkers. In 2021, Johannesen et al. analyzed the data of 13,015 statin-treated patients from the Copenhagen general population study. They showed that elevated levels of apoB and non-HDL-C were associated with an increased risk of all-cause mortality and myocardial infarction (MI), but these associations were not found with elevated levels of LDL-C [55]. Patients with discordantly high apoB compared with LDL-C (apoB above median apoB and LDL-C below median LDL-C) had a HR of 1.21 for all-cause mortality and 1.49 for MI, while those with discordantly high apoB compared with non-HDL-C had a HR of 1.21 for all-cause mortality and 0.93 for MI. Furthermore, when both apoB and non-HDL-C are discordantly high compared with LDL-C, the HR was 1.23 for all-cause mortality and 1.82 for MI. In 2021, Marston and colleagues published a prospective cohort analysis, including 389,529 primary ASCVD prevention candidates from the UK Biobank, and 40,430 statin-treated patients from the Further Cardiovascular Outcomes Research with PCSK9 Inhibition in Subjects with Elevated Risk (FOURIER) trial and the Improved Reduction of Outcomes: Vytorin Efficacy International Trial (IMPROVE-IT) [9]. They examined the individual associations of baseline apoB, non-HDL-C, and TG concentrations with incident MIs. In fully adjusted models, only apoB remained significantly associated with MI in the primary prevention cohort (adjusted HR: 1.27 per 1 SD; 95% confidence interval [CI], 1.15–1.40; *p* < 0.001). In the secondary prevention cohort, apoB was again the only biomarker found to be independently associated with MI. It was also observed that there was no longer a significant association between the ratio of TG to LDL-C (a surrogate for the ratio of TRL to LDL) and the risk of MI when the model was adjusted for apoB.

## 4. Clinical Utility of ApoB in Secondary Prevention

Secondary prevention of ASCVD generally entails increasing the intensity of statin treatment or adding a second lipid-lowering agent to achieve a ≥50% reduction in LDL-C and an LDL-C threshold of ≤1.8 mmol/L. Most recent guidelines recommend considering adding either ezetimibe or a PCSK9i in cases where the patient fails to achieve the above LDL-C targets on the maximum dose or maximum tolerated dose of statin therapy. Some have suggested that these medications should also be considered if the patient has achieved their LDL-C goal but not their apoB or non-HDL-C targets [17,18,19]. Ezetimibe and PCSK9is both tend to reduce LDL-C more than they do apoB. Add-on ezetimibe typically achieves additional LDL-C reductions of about 20% and apoB reductions of about 17%, while an add-on PCSK9i achieves additional LDL-C reductions of 50–60% and apoB reductions of only 46–53% [63,64,65,66,67]. With PCSK9i therapy, it is now possible for patients to reach remarkably low LDL-C values, below 1.8 mmol/L, but the achieved apoB is not always correspondingly low. Thus, in high-risk individuals, measuring apoB and treating to apoB targets is emphasized to ensure optimal lipoprotein-associated risk reductions. In fact, it is now clear that discordance between LDL-C and apoB, and between non-HDL-C and apoB, exists across the range of values for these parameters, suggesting that apoB should be used more broadly [43].

In 2022, Hagström et al. [42] analyzed data from the ODYSSEY treatment trial, including 18,924 patients with a recent episode of acute coronary syndrome, who had not met their treatment targets despite high-intensity or maximally tolerated statin therapy. This cohort was split into a treatment arm and a control arm, with the treatment group receiving a subcutaneous injection of the PCSK9i, alirocumab, 75 mg fortnightly, while the control group received a placebo and the baseline therapy. The investigators analyzed the risk of MACE by baseline and achieved lipid parameters at 4 months. They found that while baseline apoB held independent prognostic value when the model was adjusted for the Friedewald LDL-C, it lost significance when adjusted for the Martin/Hopkins LDL-C. Continuous baseline apoB was a more sensitive marker of risk than non-HDL-C, which had an otherwise similar linear relationship with risk. On the other hand, the benefit of 4 months’ treatment on alirocumab increased with decreasing apoB below 1.3 g/L after adjustment for LDL-C by both methods. Continuously achieved apoB had a significant linear relationship with risk, after adjustment for LDL-C and non-HDL-C, while the converse was not the case. Of note, the relationship between achieved non-HDL-C and risk was flat, with a rapidly widening confidence interval as non-HDL-C values increased, such that it crossed the HR = 1 line for most values of non-HDL-C. When tertiles of achieved apoB were cross-tabulated with tertiles of achieved LDL-C, the risk of suffering from a MACE increased with increasing apoB for each tertile of LDL-C, but there was no relationship between MACE and achieved LDL-C. 

Marston et al. [9] performed a similar analysis on 40,430 statin-treated patients from the FOURIER and IMPROVE-IT studies, who were followed up for a median of 2.5 years. The study interventions were addition of the PCSK9i, evolocumab, or ezetimibe, respectively. Achieved apoB after add-on therapy was predictive of fatal MI after adjustment for non-HDL-C, HDL-C, and TG concentrations, and clinical factors. In contrast, non-HDL-C was no longer predictive after adjustment for apoB, and TGs were not predictive after adjustment for clinical parameters, or both clinical and lipid parameters. The ratio of TG/LDL-C was also analyzed in this cohort to determine whether TRLs or LDLs have a greater association with MI. High ratios were achieved in this cohort due to LDL-C lowering, and the association line was flat up to a ratio of two, meaning that neither lipoprotein type poses a higher risk of MI than the other. These findings were all consistent in sensitivity analyses using selected subgroups.

## 5. Assay Standardization

While many guidelines have now acknowledged that apoB is the most accurate lipid marker of ASCVD risk and response to therapy [17,18,19], and the European guideline has endorsed these assays as well-standardized and accurate and acknowledged that they are superior to the measurement or calculation of LDL-C and non-HDL-C [18], there are still those that question the reliability of apoB measurements [19]. To laboratorians, “accuracy” is a measure of how close a measurement or prediction is to the “truth”. In the case of analytical accuracy, this “truth” is determined using the “gold standard” or reference method, while in the case of diagnostic accuracy, the “truth” is the true diagnosis or prognosis. The accuracy of a lipid-associated risk prediction is a composite of the biological relationship of the actual plasma concentration of the lipid or the lipoprotein with risk, and the ability of the assay to provide a true measurement of that concentration. The findings in the studies mentioned above represent this composite accuracy as they evaluated the relationships of the lipids with risk using measurements obtained through employing the available assays with their current analytical performance. It is therefore perplexing that this evidence has been discounted, particularly by clinicians, due to the supposed lack of standardization of these apoB assays, when it is these same assays that proved to be accurate across various manufacturers in these clinical trials.

ApoB may be routinely measured on automated clinical chemistry analyzers using immunoturbidimetry or immunonephelometry. These same assay principles are used to accurately measure other proteins, such as C-reactive protein, immunoglobulins, and transferrin, in routine clinical chemistry laboratories. The World Health Organization (WHO) and International Federation of Clinical Chemistry (IFCC) have established a standardization program and a secondary reference material (SP3) for apoB, as detailed by Marcovina et al. [68], and evidence from international proficiency testing programs suggests that apoB assays perform well [69,70]. The EAS and the European Federation of Clinical Chemistry and Laboratory Medicine (EFLM) Joint Consensus Initiative, in fact, reported that apoB assays have better analytical performance than LDL-C and non-HDL-C estimation procedures [71]. While apoB has not yet been standardized to a pure, higher order reference material, the same is true for the other lipoprotein markers. Furthermore, as lipoproteins are heterogeneous, polydisperse particles, they cannot be isolated as pure substances and will likely never be truly “standardized”, according to the International Organization for Standardization standard 17511:2020 requirements. In contrast, the amino acid sequences recognized in apoB assays are well defined. This has enabled the recent development of a primary reference method to accurately measure apoB using mass spectrometry [72], which should be implemented soon to further improve the standardization of apoB.

In addition to its better standardization, apoB is largely unaffected by the fasting or non-fasting states. ApoB assays are also unaffected by high degrees of lipemia [71], and unit conversions are within the weight-based metric system (i.e., multiples of 10) and no further calculations are, therefore, required. In contrast, calculation of LDL-C is fraught with controversy. Most laboratories still use the Friedewald equation when TG ≤ 4.5 mmol/L and variably do not calculate it or use one of the alternative equations when TG > 4.5 mmol/L. This is due to the fact that the Friedewald equation assumes a fixed ratio of TG to VLDL-C, and the error inherent in this assumption exceeds acceptability when TG > 4.5 mmol/L. Owing to problems with lipoprotein specificity, direct LDL-C measurements do not necessarily improve the accuracy of LDL-C determinations, as shown by Miller et al. [73]. While non-HDL-C calculation escapes the issue of the TG conversion factor, and thereby avoids this error in its calculation [74], it still includes HDL-C measurement, which is affected by elevated TGs and other matrix effects that are common in dyslipidemias [71]. Furthermore, the uncertainty of non-HDL-C determination is affected by the additive uncertainties in the TC and HDL-C measurement procedures, and it is also affected by unit conversions between the standard international and mass units.

## 6. Utility of ApoB versus Non-HDL-C as ASCVD Risk Markers

The inclusion of cholesterol on all apoB-containing lipoproteins in non-HDL-C helps to partially correct for the underestimation of risk by LDL-C in hypertriglyceridemia. In addition, non-HDL-C may be calculated from the standard lipid panel at no additional cost. For these reasons, in the Canadian guidelines, routine calculation of non-HDL-C is recommended [17]. The EAS and the EFLM Joint Consensus Initiative also recommends non-HDL-C calculation for all patients [71]. Likewise, the US Multisociety guidelines allude to equivalence between non-HDL-C and apoB and promote non-HDL-C calculation in place of apoB when LDL-C is inaccurate [19]. 

**Figure 2:**
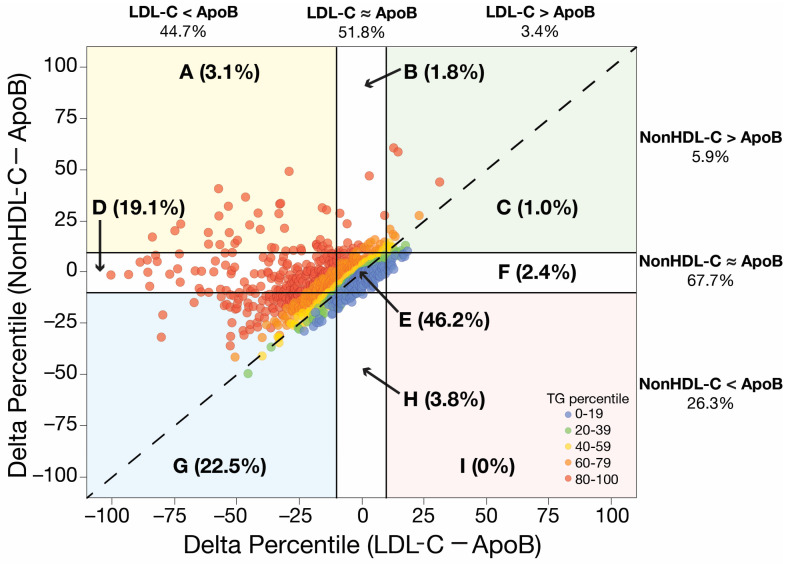
Discordance between LDL-C and non-HDL-C, and apoB. Lipid profile test results (N = 1121) fromNHANES (2005–2016) for individuals aged 18–75 years (mean age = 62; 58% male), treated with lipid-lowering medication, and with LDL-C levels < 100 mg/dL (mean LDL-C = 76 mg/dL) were used to calculate the difference in percentile units between LDL-C and apoB (*x*-axis), and between non-HDL-C and apoB (*y*-axis). Concordant LDL-C (Sectors B, E and H) and non-HDL-C results (Sectors D, E and F) were defined as being within ±10 percentile units of the apoB percentile value. Individual data points are color coded with the percentile of the triglyceride values. The percentage of the total population in each individual sector (next to sector designation) or combined sectors (column and row headings) is indicated. LDL-C was calculated using the Friedewald equation. White background: concordant sectors; colored background: discordant sectors.

As shown in Figure 2, although non-HDL-C is less discordant with apoB than is LDL-C, it frequently results in a different risk assessment than apoB. In approximately one-third of individuals in NHANES on a lipid-lowering medication, concentrations of non-HDL-C and apoB differed by more than ±10% on a population percentile basis. Although the use of non-HDL-C in hypertriglyceridemic patients correctly raises the risk assessment in most of these patients, it sometimes leads to an overestimation of risk, particularly in those with the highest triglycerides (Figure 2: Sector A). In other cases of hypertriglyceridemia, risk is still underestimated by non-HDL-C (Figure 2: Sector B). Again, this relates to the complicated relationship between particle number, size, and lipid composition, as well as analytical limitations, which often lead to a disconnect between these parameters, and wide dispersion around the regression line between non-HDL-C and apoB. 

Numerous discordance studies, Mendelian randomization studies, and the prospective cohort studies already discussed showed that non-HDL-C is not equivalent to apoB as a marker of risk. Whether or not non-HDL-C is adequate, however, remains controversial. The main arguments in favor of its adequacy are its stronger correlation and reduced discordance with apoB compared with those of LDL-C, essentially indicating that it is “good enough”. Proponents of apoB raise the point that population-derived statistics, such as correlation coefficients, are less meaningful for the individual. The residual, or difference between the observed and expected apoB after linear regression of apoB on non-HDL-C, provides additional risk information [54], and shows that non-HDL-C is not always adequate for individual patients. In the large meta-analysis discussed above, Sniderman et al. found that treating to apoB targets could prevent 500,000 more cardiovascular events among US adults over 10 years than treating to non-HDL-C targets [62]. This, and the clinical trials discussed above, partially address the argument that there is a lack of evidence indicating that treating to apoB or non-HDL-C targets improves clinical outcomes [71]. While direct outcomes studies remain desirable, the evidence for LDL-C targets is also not robust [45].

The Canadian and European guidelines provide treatment target values for both non-HDL-C and apoB [17,18], whereas the US Multisociety guideline provides targets only for non-HDL-C [19]. In the Canadian guideline, the target values provided for both non-HDL-C and apoB are percentile equivalents of the recommended LDL-C targets [17]. The European guideline provides apoB treatment targets based on a study of 1154 diabetic patients treated with atorvastatin or a placebo [18]. They performed linear regression of the achieved apoB on the achieved LDL-C to provide equivalent achieved apoB values to the target LDL-C values in the treatment arm of this cohort [75].

The US and European guidelines provide non-HDL-C targets that are a fixed 0.8 mmol/L greater than the LDL-C targets. This is based on an ideal TG value of 1.7 mmol/L and assumes a fixed ratio of TG to VLDL-C of 2.2, as per the Friedewald formula [49]. This is not ideal, as we know that this ratio is not fixed. Given that this is one of the major problems with LDL-C calculations, it is perplexing that this spurious ratio is still included in the determination of non-HDL-C treatment targets.

It has also been argued that apoB does not substantially improve the accuracy of risk prediction models that already include other risk factors. This argument no longer seems to hold once it is appreciated that the order in which markers are added to a model affects the incremental predictive value assigned to that marker [54,76,77], and several groups have demonstrated an impressive predictive value of apoB in risk models [54,78]. Aside from its role as a secondary risk marker, the European guideline states that apoB may be used as the primary marker for screening, diagnosis and management, where available. It also recommends apoB measurement in patients with hypertriglyceridemia, obesity, diabetes, the metabolic syndrome, or very low LDL-C, and states that it may be preferred over non-HDL-C in these cases [18]. In Canada, apoB is an insured laboratory test in all but one province and, while apoB is explicitly acknowledged as the better marker, it is left to clinicians to choose between apoB and non-HDL-C, depending on their level of comfort with each measure, the availability of testing, and concern regarding discordance between the markers [17].

In our view, non-HDL-C should be reported for all patients. Like apoB, it is superior to LDL-C as it is a more accurate proxy for atherogenic lipoprotein concentration. Also, it does not involve any additional cost and, provided the TG concentration is not high enough to affect the HDL-C assay, it is not affected by the non-fasting state [79]. ApoB, however, is superior to non-HDL-C and is cost-effective [80]. In the current paradigm of precision medicine, it is likely that the value of the additional precision of apoB will ultimately result in its adoption as the primary risk marker for ASCVD.

## 7. Conclusions

The preponderance of evidence indicates that apoB is the superior biomarker for ASCVD prevention compared to other lipid and lipoprotein-related measures. Its measurement is now adequately standardized, and it can be measured accurately and precisely using automated assays on clinical chemistry analyzers [68,71]. Based on the College of American Pathology proficiency surveys, however, apoB assays are not widely offered by clinical laboratories in the US, and this may also be the case elsewhere. This poor availability reflects low demand for the assay, which may be attributed to several barriers, including the guideline recommendations, a lack of familiarity of clinicians with interpretation of apoB results, their value, and the cost of testing.

International guidelines are gradually promoting apoB measurement in specific contexts, and some have recommended initial assessment cut-offs and treatment targets (Table 1). With the ongoing development of precision medicine approaches, and with more widespread acknowledgement of and participation in the WHO/IFCC standardization program, further guidance on the use and interpretation of apoB may be expected. In the interim, clinical laboratories may illustrate the importance of apoB, and assist clinicians in interpretation, by converting apoB results to percentile equivalent LDL-C results [43]. This may also serve to improve familiarity with apoB values. It is imperative for laboratorians to continue to educate clinicians on the validity and standardization of the apoB assays that are routinely available.

Another challenge is the current cost of apoB assays. It was already shown that adding apoB to the lipid panel would not substantially increase overall costs, due to its clinical effectiveness [80]. Given the much higher costs of drug therapy, particularly for the newer agents, and the even higher costs related to inadequate treatment of high-risk individuals, treating to apoB targets is the most reliable and cost-effective strategy for mitigating lipid-associated residual risk [9,42]. Most US insurance companies will reimburse apoB in high-risk individuals, and it is an insured test throughout most of Canada. With more demand from clinicians and patients, and with advocacy from clinical chemists, it may be possible to reduce the reimbursement rate for apoB, making it more financially accessible.

It was predicted that the next paradigm shift in ASCVD risk prediction in this era of precision medicine will involve individualized calculations of the potential for net benefit from treatment [81]. Although a step in the right direction, the pooled cohort equations applied in the US guideline are known to regularly overestimate risk, and population-based risk scores often show a poor level of specificity when applied on an individual basis [82,83,84]. Future individualized risk assessment will likely include currently known risk markers and may include new, emerging risk markers, such as markers of HDL dysfunction and LDL oxidation, as well as genetic markers. Ideally, an individualized net treatment benefit prediction will also include an estimation of the risk of adverse events secondary to treatment [81]. Based on our analysis of the literature, replacement of LDL-C with apoB is a step that should be taken now in managing lipid-lowering therapy, and perhaps eventually for screening in primary prevention once the infrastructure is in place for its more widespread use.

## Figures and Tables

**Table 1:** ApoB lipoprotein cut-offs and treatment targets.

ApoB Lipoprotein Cut-Offs to Initiate Statins in the 2021 Canadian Guideline [18]			
Framingham risk score	LDL-C	ApoB	Non-HDL-C
<10%	≥5.0 mmol/L	≥1.45 g/L	≥5.8 mmol/L
5–9.9% with other CV risk factors	≥3.5 mmol/L	≥1.05 g/L	≥4.2 mmol/L
10–19.9%	≥3.5 mmol/L	≥1.05 g/L	≥3.5 mmol/L
**Statin Treatment Targets Recommended in the 2021 Canadian Guideline** [18]			
Statin indication	LDL-C	ApoB	Non-HDL-C
FH or genetic dyslipidemia	<2.5 mmol/L	<0.85 g/L	<3.2 mmol/L
Intermediate or high risk, DM2, and CKD	<2.0 mmol/L	<0.8 g/L	<2.6 mmol/L
ASCVD for ezetimibe	<1.8 mmol/L	<0.7 g/L	<2.4 mmol/L
ASCVD for PCSK9i	≤2.2 mmol/L	≤0.8 g/L	≤2.9 mmol/L
**Treatment Targets Recommended in the 2019 European Guideline** [19]			
Risk group	LDL-C	ApoB	Non-HDL-C
Moderate	<2.6 mmol/L	<1.0 g/L	<3.4 mmol/L
High	<1.8 mmol/L	<0.8 g/L	<2.6 mmol/L
Very High	<1.4 mmol/L	<0.65 g/L	<2.2 mmol/L

Abbreviations: CV, cardiovascular; FH, familial hypercholesterolemia; DM2, diabetes mellitus type II; CKD, chronic kidney disease; ASCVD, atherosclerotic cardiovascular disease; and PCSK9i, proprotein convertase subtilisin/kexin type 9 inhibitor.

## Data Availability

Not applicable.
